# Supplementary material for: Modulation of antigen discrimination by duration of immune contacts in a kinetic proofreading model of T cell activation with extreme statistics
Source: PLoS Comput Biol. 2023 Aug 30;19(8):e1011216. doi: 10.1371/journal.pcbi.1011216 (PMC10497171; doi:10.1371/journal.pcbi.1011216)
Supplement: Attachment — Submitted filename: ResponseToReviewers.pdf [file pcbi.1011216.s002.pdf]

Response to referees

Reviewer #1

Overall Comment: *The computational and mathematical results in this paper contribute a novel, unappreciated role for the lifetime of the cell-cell contact. The authors clearly and rigorously give background for the need of their new model, both biologically and mathematically. The idea of terminating cell-cell contacts leading to one hundred percent accuracy is an unexpected and powerful result.*

Response: Thank you for your careful reading of the paper and positive remarks on our results. We have addressed all your comments below.

Specific Comments:

1. *For some model parameters, estimates from previous literature are discussed, but not the number of KP steps. Since this is such a key parameter, the authors should include discussion of other estimates of the number of KP steps. From a molecular structural viewpoint: how many phosphosites are there per TCR? How many ITAMs? This gives a rough upper bound on the number of steps. From a whole-cell viewpoint: Pettman et al. 2021 eLife also contains related results.*

   Response: We have added a paragraph to the introduction discussing this point. Specifically, we have discussed the potential role of ITAMs and ZAP-70 in determining the possible number of KP steps.

2. *Figure 4 and the subsection on energy is unclear. Indeed, it seems the results shown in the figure contradict the headline: There are regions of parameter space where reducing contact lifetime allow the consumption of less energy, while increasing the accuracy.*

   Response: The explanation for this observation is that increasing just one parameter (e.g. n_{KP}) while keep others fixed (e.g. τ or n_e) can decrease accuracy. Improved maximum accuracy from additional KP steps can only be realized in our model through increased contact duration. We have added a paragraph of discussion on this point.

3. *Is the fact that $T_{1,n_{self}}$ is a Weibull random variable used in the simulations? In other words, do the simulations sample from a Weibull to accelerate simulation time? If so, add details in the model section.*

   Response: It is not used in the simulations. and we just verify that $T_{1,n_{self}}$ approaches a Weilbull random variable as $n_{self} \to \infty$. This limiting case ($n_{self} \gg 1$) is utilized only to provide additional validation for both our analytical results and simulations.

4. Typo: First paragraph of results: $\sigma \geq 10$ should be replaced with $n_{KP} \geq 10$

   Response: Fixed this.

5. *Figure 2B appears to have strange bump in the $\sigma = 15$ curve near the peak. Is this a rendering error, or a numerical artefact?*

   Response: This was indeed an artefact that we have now fixed.

Reviewer #2

Overall Comment:

I think this is a very interesting paper that deserves to be published in PLOS Comp Bio. The authors address an important biological problem which has been studied with theoretical models for quite some time, and yet the authors present an important new perspective that prior "steady-state" analyses seem to miss. My main critiques are the following more technical questions which I think the authors should address. I think most of these critiques could be addressed by a clearer description of the model in the main text.

Response: Thank you for your positive remarks on our work and for your helpful comments. We have addressed your comments below and believe the presentation of the manuscript is much improved.

Specific Comments:

1. *Page 6 of the main text says that the formula for the accuracy* Γ *is given in the supplement. Where is the formula for* Γ *in the supplement? I couldn't find it.*

   Response: Added more clarity on this definition of Γ and its derivation. The definition $\Gamma = \mathbb{P}(TP) + \mathbb{P}(TN)$ is given in equation (3) with the relevant statistics given in equation (2). The practical calculation of Γ requires us to determine the distribution of $T_{1,n}$ for $n = 1$ (shown explicitly in Sec. 1.1 of supplement) and for $n \gg 1$ (approximated in Sec. 1.2 of supplement).

2. *In some instances, the authors find optimal values of* τ *are on the order of* $\tau = 10^5$. *Looking at Table 1,* τ *is said to have units of seconds, in which case this is* $\tau \approx 30$ *hours which I assume is unphysiological. By saying that* τ *has units of seconds, do the authors merely mean that it has units of time, and the authors have simply scaled time for that* k_1, k_p, *and* k_{-1} *are all unity (along the lines of section 5 in the supplement)? If so, can the authors estimate the values of these rates (from the literature) to translate their optimal values of* τ *into real time values? Along these lines, are there available estimates for* σ*?*

   Response: In some of the earlier works by Kersh et. al. (e.g. 1998 Immunity), the parameter σ could be approximated from the experimental data. In some cases, a 5-fold difference in dissociation rate ($\sigma = 5$) could result in large differences in T cell activation sufficient to differentiate an agonist antigen from a weak antagonist. However, there is some degree of uncertainty about the actual T cell environment. For example, when our model predicts near-perfect accuracy for $\sigma = 5$, we have also explicitly modeled the self and agonist antigen populations. It is not clear in experiments Kersh et. al. the population of weak antagonists and agonists present in the T cell/APC contact and it would be interesting to compare our model with experimental data in which these variables are controlled. Furthermore, Pettman et. al. (2021 eLife) have recently shown that this "very good" (low σ) antigen discrimination observed in early experiments may be an artefact. So it may be necessary to re-evaluate any possible values for σ.
   In terms of obtaining real-time optimal τ values, this would indeed be possible if we were to use biologically relevant rates, KP steps, and σ estimated from the literature. However, there is a similar problem here. The optimal τ^* we would find in this case would be specific to some self and agonist antigen populations. Thus, we would also need, at the very least, an estimate of the distribution of these quantities for the T cell environment in question. Furthermore, the number of effective KP steps is still under investigation. In the past, there were many phosphorylation

sites being investigated as potential KP steps. But now only a small number of these sites appear to be relevant to the type of antigen (Voisinne, 2022 Nature Immunology). Other structures, such as microvilli on the surface of the T cell, could influence the temporal dynamics of the T cell response.
Our main contribution is a method to evaluate the relevance of the cellular contact duration in T cell accuracy. A potential set of experiments could be to systematically vary cellular contact duration, in both agonist positive and agonist negative APC populations, and derive accuracy curves with respect to the cellular contact duration. This could establish if such a thing as optimal contact duration exists. It may also demonstrate that the mechanism of antigen discrimination operates, at least in part, in response to first-passage activation events.

3. *Are the units of k_1 correct? Table 1 says that it is an inverse time, but looking at equation (14a) in the supplement, it seems like it is a bimolecular reaction rate and thus has units of $1/(concentration * time)$ assuming L_T has units of concentration. It says L_T is the total population of ligands, so the units are simply the raw number of ligands? Perhaps R_T, L_T should be added to Table 1?*

   Response: k_1 is indeed a bimolecular reaction rate, and would make more sense to have units $1/(concentration * time)$ in a continuous mass action model. This may just be confusion in perspective or semantics, but equation (14) in the supplement is technically not an approximation of the mass action model. Instead, it is an approximation for the extreme first passage time (equation 14e) from a discrete stochastic system. By treating $R(t)$ and $L(t)$ as dimensionless counts, this gives units for dA/dt to be probability/unit time, which can be interpreted as a hazard function.

4. *The supplement presents a few different approximations. Which one of these approximations is used to plot Γ in the main text figures?*

   Response: In the single agonist case ($n = n_{ag} = 1$), we use the density given in equation (12) of the supplement. In the large self antigen population ($n = n_{self} \gg 1$), we use equation (14) of the supplement. The calculation of Γ then follows from equations (3) and (2) in the main text. We have added a sentence in the main text clarifying this. In particular, we do not use the Weibull approximation in any of our main results - it is only included for validation in the $n \gg 1$ limit.

5. *I think the ODE approximation in the supplement should be clarified. In particular, I don't understand the "nonlinear binding rate $K_1(R(t), L(t))$" mentioned at the top of page 5 of the supplement.*

   Response: This is the bi-molecular (mass-action) binding rate at which available ligands ($L(t)$) and receptors ($R(t)$) interact. We have added more clarifying text to the supplement.

6. *The self-activation time is defined as the minimum of $t_1, t_2, \ldots t_{n_{self}}$. Are these random variables independent and identically distributed (i.i.d.)? I think they are, but then I don't understand the following statement from the Supplement: "A source of numerical error in applying this result [3] to a large antigen population in the FRAM is that the first passage activation of receptors are not i.i.d., since the propensity for binding events is $K_1 = k_1RL$." I don't understand this sentence. I think this sentence should be clarified and the model assumptions (namely of independence or dependence) should be clarified in the main text.*

Response: They are not i.i.d.. This is the reason the approximation displayed in equation (14) of the supplement is necessary for the extreme statistic, rather than the simpler case of finding the minimum of i.i.d. samples. The reason the receptor activation times are not i.i.d. is that the binding rate depends on both the populations of unbound receptors and the population of unbound ligands at time t. This means the binding propensity of a realization of the stochastic process at time t is $K_1 = k_1 R(t) L(t)$, where $R(t)$ and $L(t)$ is the number of unbound receptors and ligands at time t. We have added more clarification to this in the supplement.

7. On page 4 of the supplement, there are some references to equation (13) which should be references to equation (12) (for example, it says "The integral (13) is readily..."

   Response: Fixed this.
